# KIF23 activated Wnt/β-catenin signaling pathway through direct interaction with Amer1 in gastric cancer

**DOI:** 10.18632/aging.103146

**Published:** 2020-05-04

**Authors:** Yi Liu, Hui Chen, Ping Dong, Guohua Xie, Yunlan Zhou, Yanhui Ma, Xiangliang Yuan, Junyao Yang, Li Han, Lei Chen, Lisong Shen

**Affiliations:** 1 Department of Clinical Laboratory, Xinhua Hospital, Shanghai Jiao Tong University School of Medicine, Shanghai 200092, China; 2 Department of General Surgery, Xinhua Hospital, Shanghai Jiao Tong University School of Medicine, Shanghai 200092, China

**Keywords:** gastric cancer, KIF23, Amer1, Wnt/β-catenin signaling pathway, cell growth

## Abstract

Increased expression of the kinesin family member 23 (KIF23) has been verified in gastric cancer (GC) and its upregulation contributes to cell proliferation. Even though, the role of KIF23 has not been fully elucidated in GC, and the mechanisms of KIF23 as an oncogene remain unknown. To further identify its potential role in GC, we analyzed gene expression data from GC patients in GEO and TCGA datasets. KIF23 was upregulated in GC, and increased expression of KIF23 correlated with poor prognosis. Importantly, KIF23 inhibition not only suppressed GC cell proliferation, tumorigenesis, but also migration and invasion, and arrested the cell cycle in the G2/M phase. Mechanistic investigations confirmed that KIF23 activated the Wnt/β-catenin signaling pathway by directly interacting with APC membrane recruitment 1 (Amer1). Furthermore, KIF23 exhibited competitive binding with Amer1 to block the association of Amer1 with adenomatous polyposis coli (APC), thus relocating Amer1 from the membrane and cytoplasm to the nucleus and attenuating the ability of Amer1 to negatively regulate Wnt/β-catenin signaling, resulting in activation of this signaling pathway. Collectively, our findings demonstrated that KIF23 promoted GC cell proliferation by directly interacting with Amer1 and activating the Wnt/β-catenin signaling pathway.

## INTRODUCTION

The human KIF23 protein, also known as MKLP1, is a nuclear protein that localizes to the interzones of mitotic spindles and acts as a plus-end-directed motor enzyme that moves antiparallel microtubules in vitro [1]. Previous studies have indicated that KIF23 is a key regulator of cytokinesis [2]. Dysfunction of KIF23 results in incomplete cytokinesis and formation of binucleated or multinucleated cells, which are hallmarks of cancer [3]. Owing to their specific effects on mitosis, inhibitors of mitotic kinesin might have fewer side effects than other targeting agents currently used in the clinic. Yutaka et al. reported that deletion of KIF23 suppressed glioma proliferation, leading to the formation of large cell bodies with two or more nuclei [1]. Kato T et al. verified that KIF23 was upregulated in lung cancer and predicted a poor clinical outcome [4]. Recently, Xiaolong Li found that KIF23 upregulated in GC and silencing KIF23 suppressed cell proliferation [5]. However, to date, the mechanisms of KIF23 in cancer remain unknown.

Dysregulation of the Wnt/β-catenin signaling pathway, a critical developmental signaling pathway, is strongly implicated in the pathogenesis of many types of cancer [6–8]. Perturbation of the Wnt/β-catenin signaling pathway can promote the initiation and progression of GC and has been linked to aggressive tumor behavior [9]. Abnormal activation or mutations of key proteins are involved in the stabilization of β-catenin and, in turn, activation of transcription [10, 11]. Major et al. demonstrated that APC membrane recruitment 1 (Amer1), also known as WTX (Wilms’ tumor suppressor X chromosome), formed a complex with β-catenin and APC (adenomatous polyposis coli) to promote β-catenin ubiquitination and degradation, which antagonized the Wnt/β-catenin signaling pathway [12]. Tumors lacking this tumor suppressor exhibited a mesenchymal phenotype characterized by activation of the Wnt/β-catenin signaling pathway [13].

Here, we demonstrate that KIF23 is significantly upregulated in GC tissues, and increased expression of KIF23 promotes GC cell cycle progression by targeting the G2/M phase. In terms of mechanism, KIF23 competitively binds with Amer1 to activate the Wnt/β-catenin signaling pathway.

## RESULTS

### High expression of KIF23 predicted the poor prognosis and its carcinogenesis in GC

**Figure 1:**
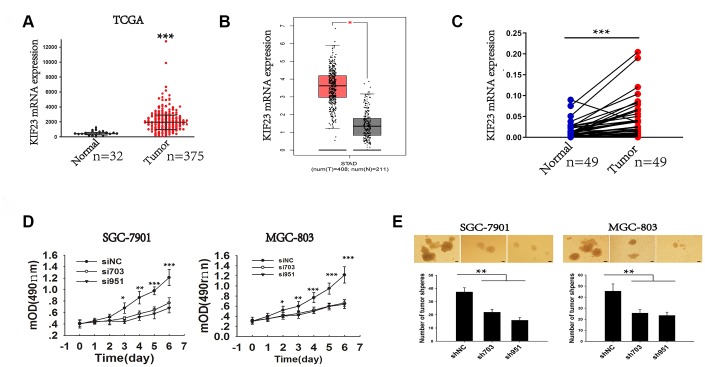
**Analysis of the elevated expression and role of KIF23 in GC.** (**A**) TCGA dataset analysis of KIF23 gene expression in normal and tumor tissues. (**B**) The expression of KIF23 in gastric cancer was analyzed by using an online tool GEPIA. (**C**) Q-PCR analysis of KIF23 gene expression in 49 pairs of GC and matched tissues from our own hospital. (**D**) MTT cell viability assay was performed in SGC-7091 and MGC-803 cells with KIF23 knockdown at different days. (**E**) Deletion of KIF23 to observe its effect on tumor sphere formation capacity in SGC-7901 and MGC-803 cells. Representative photographs of the tumor spheres and the statistical analysis are shown. Data are the means ± SEMs of three independent experiments. * p < 0.05, ** p < 0.01, *** p < 0.001 vs. control.

To further verify the role of KIF23 in GC pathogenesis, two Gene Expression Omnibus (GEO) (GSE2685 and GSE65801) and The Cancer Genome Atlas (TCGA) datasets were chosen for evaluation of KIF23 mRNA levels in GC and normal tissues. The results of datasets showed that KIF23 was significantly upregulated in GC tumors compared with normal tissues, especially in proliferative GC samples (Figure 1A, 1B and Supplementary Figure 1A, 1B). We also analyzed KIF23 levels in GC and normal tissues collected from Xinhua Hospital, and the results were consistent with the dataset analysis (Figure 1C). Similarly, assessment via immunohistochemistry (IHC) revealed that KIF23 was upregulated in GC tumors compared with matched adjacent histologically normal tissues (MN) and histologically normal tissues (NN). In addition, KIF23 expression was markedly upregulated in tumors from patients with phase III GC compared with samples from phase II and I patients (Supplementary Figure 1C and 1D). And higher KIF23 expression in GC patients was correlated with shorter survival times, which was consistent with the analysis of publicly available datasets (http://kmplot.com/analysis/index.php?p=service&cancer=gastric) (Supplementary Figure 1E and 1F).

To elucidate the role of KIF23 in gastric carcinogenesis, we first analyzed KIF23 expression among 6 GC cell lines, and high KIF23 levels were observed in SGC-7901 and MGC-803, but low KIF23 levels were observed in MKN-45 and BGC-823 (Supplementary Figure 2A and 2B). Then, we employed siRNAs and shRNAs to silence KIF23 expression in SGC-7901 and MGC-803 (Supplementary Figure 2C–2F). KIF23 knockdown significantly suppressed the growth rate of the two GC cell lines (Figure 1D) and inhibited colony and tumor sphere formation by these cells (Supplementary Figure 3A and Figure 1E); however, KIF23 knockdown had no effect on cell apoptosis (Supplementary Figure 3G). Furthermore, knockdown of KIF23 expression significantly suppressed the in vivo tumorigenicity of SGC-7901 and MGC-803 cells after subcutaneous injection of the cells into nude mice, as demonstrated by reduced tumor size and weight (Supplementary Figure 3B). In formed tumor tissues, significantly reduced expression of KIF23 was observed after KIF23 knockdown (Supplementary Figure 3C), and the tumors formed in the shRNA-KIF23 group were noninvasive or well-encapsulated tumors (Supplementary Figure 3D). Besides, the KIF23 knockdown group exhibited lower wound healing, migratory and invasive capacities than the corresponding siNC group (Supplementary Figure 3E and 3F). On the other hand, KIF23 overexpression promoted GC cell growth, migration and invasion (Supplementary Figure 4). Our data strongly suggest that elevated KIF23 expression induces enhanced oncogenic potency and is associated with the progression of GC.

### Inhibition of KIF23 arrested cell cycle in the G2/M phase

**Figure 2:**
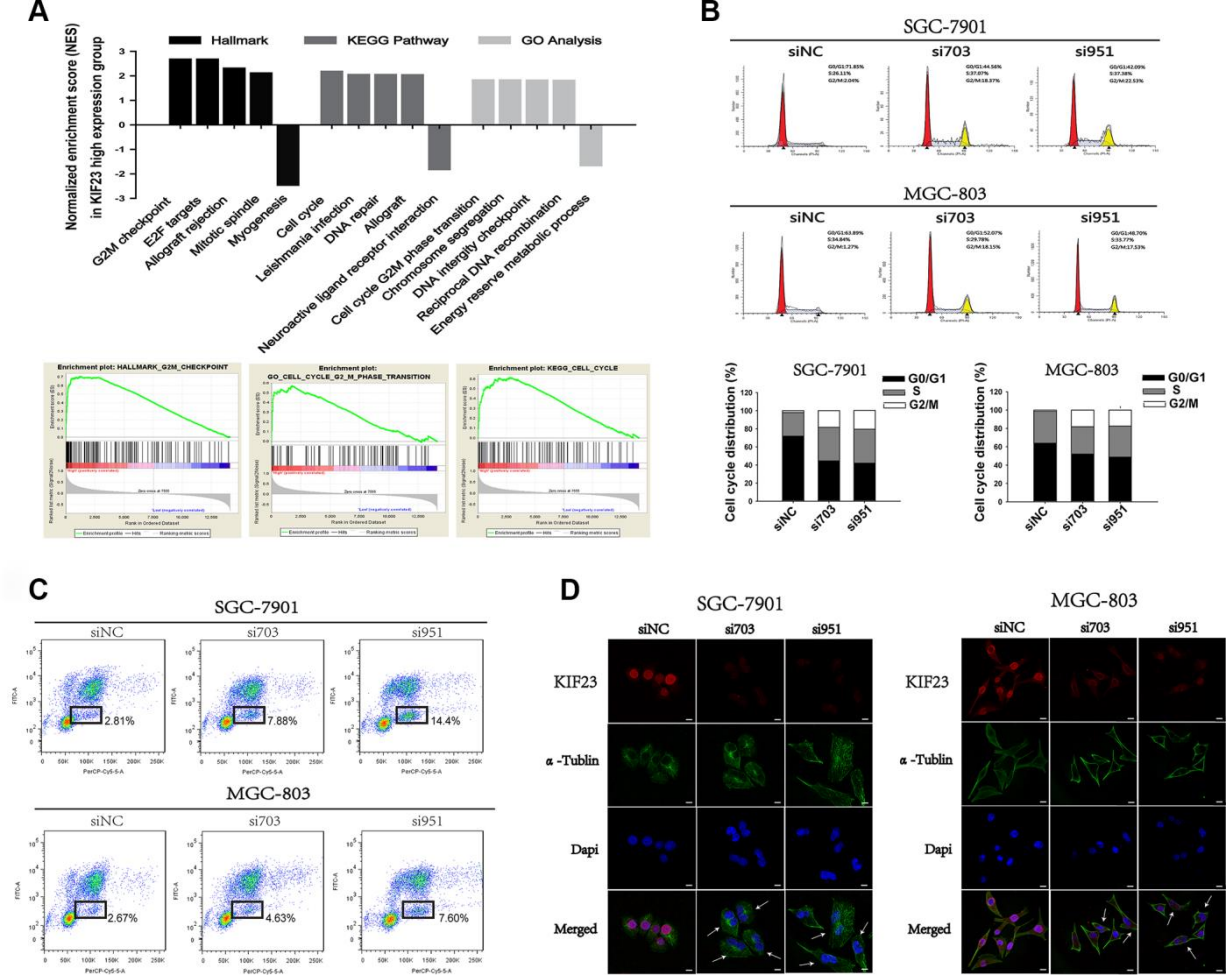
**KIF23 knockdown suppressed cell cycle progression of GC cells.** (**A**) A public dataset (GSE65801) was divided into two groups—a high KIF23 expression group and a low KIF23 expression group—and the normalized enrichment score (NES) of three gene set categories was calculated by gene set enrichment analysis (GSEA). (**B**) Cell cycle and (**C**) BrdU incorporation assay analysis of GC cells transfected with siRNAs against KIF23. The percentages of the cell subpopulations at different stages of the cell cycle were statistically analyzed. (**D**) Immunofluorescence images showed the morphology of GC cells after being transfected with siRNAs.

To explore the mechanism by which KIF23 regulates cell proliferation, we carried out gene set enrichment analysis (GSEA) using public datasets (GSE65801), and the results showed that high KIF23 expression was positively associated with cell cycle progression (Figure 2A). Subsequently, we monitored cell cycle progression using FACS after KIF23 knockdown. Interestingly, a higher proportion of cells transfected with siRNA-KIF23 were arrested at the G2/M phase of the cell cycle than that of cells transfected with siNC (Figure 2B and2C). This finding suggested that KIF23 may participate in regulating cell cycle progression in GC cells. Furthermore, the effect of KIF23 knockdown on the shapes of these cells was examined by confocal microscopy. Typical fluorescent images of the stained cells are shown in Figure 2D. An increased number of KIF23 siRNA-treated GC cells exhibited large cell bodies with two nuclei. The KIF23 siRNA-treated GC cells may not have been able to complete cytokinesis properly; therefore, these cells were arrested in the G2/M phase of the cell cycle.

### KIF23 promoted cell growth via activation of the Wnt/β-catenin signaling pathway and its relationship with PRC1

**Figure 3:**
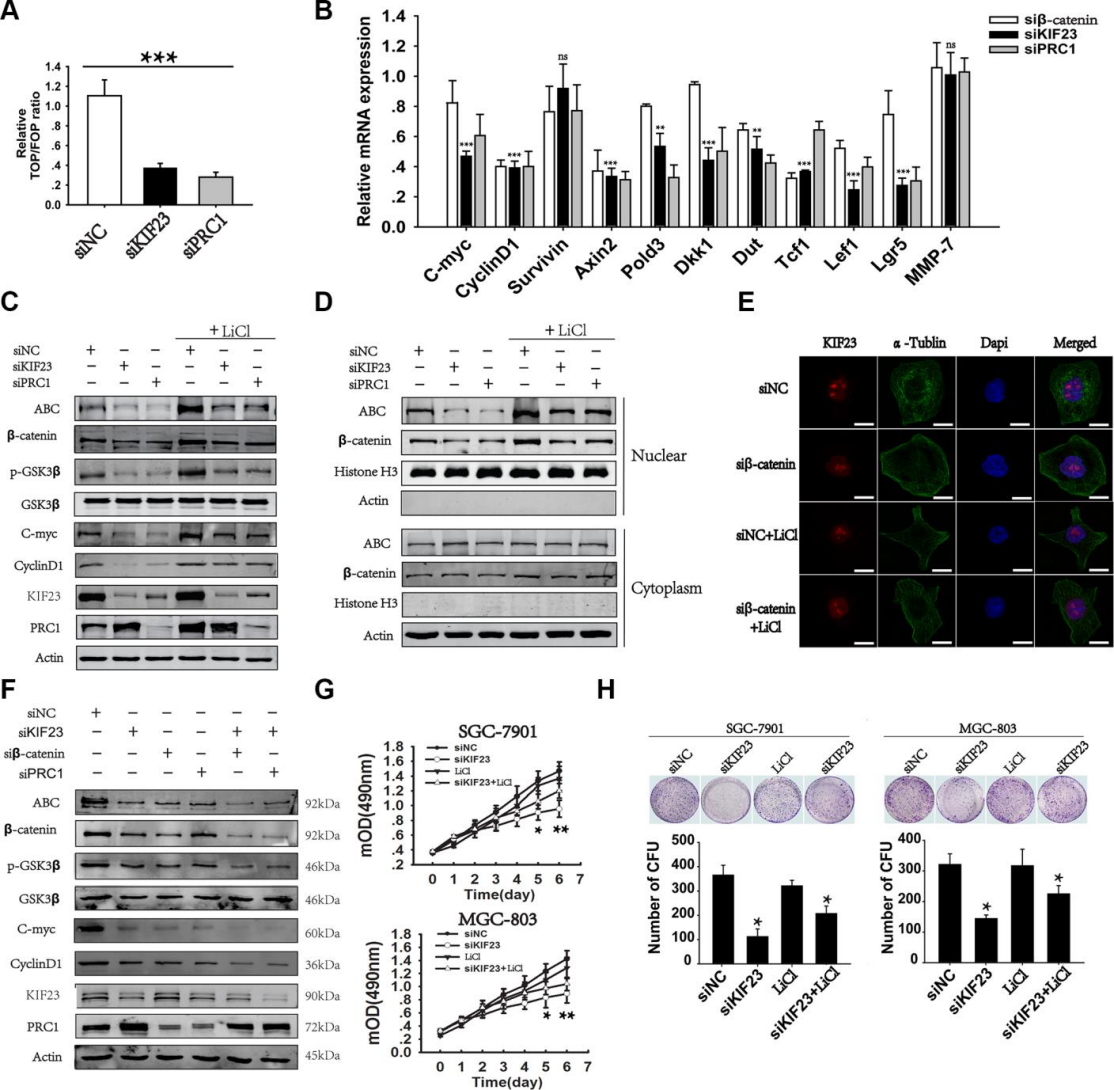
**Loss of KIF23 impaired the Wnt/β-catenin signaling pathway in GC.** (**A**) TCF luciferase reporter plasmid and its mutant plasmid were constructed and transfected into MGC-803 cells. Changes in endogenous Wnt/TCF reporter activity in MGC-803 cells after silencing of KIF23 or PRC1 were analyzed. (**B**) Q-PCR analysis of the effects of KIF23 and PRC1 silencing on 11 Wnt target genes in MGC-803 cells. (**C**) Western blot analysis of the levels of KIF23, β-catenin activation and Wnt targets in MGC-803 cells treated with siNC, KIF23 siRNA (siKIF23) or PRC1 siRNA (siPRC1) with or without LiCl for activation of the Wnt/β-catenin signaling pathway overnight. (**D**) Nuclear and plasma proteins were extracted, and Western blot analysis of β-catenin activation was performed to reveal the distribution of β-catenin in nuclear and cytoplasmic fractions (Histone H3: nuclear protein marker, actin: cytoplasmic protein marker). (**E**) Immunofluorescence staining for nuclear β-catenin after siRNAs application. β-catenin was labeled in red, and the cytoskeleton protein was labeled in green. (**F**) Western blot analysis of β-catenin activation and Wnt targets after rescue of PRC1 levels with siRPC1. Actin was used as the loading control. Cell growth (**G**) and colony formation assays (**H**) were performed after KIF23 knockdown and LiCl activation. All experiments were repeated at least three times. Statistically significant differences are indicated.* p < 0.05, ** p < 0.01, *** p < 0.001; ns, no significant difference.

Oncogenic activation of the Wnt/β-catenin signaling pathway is common in GC. To investigate the potential role of KIF23 in the Wnt/β-catenin signaling pathway, we first determined the effect of KIF23 on T cell factor (TCF) activity in GC cells. Unexpectedly, KIF23 knockdown significantly inhibited TCF luciferase reporter activity in MGC-803 cells. TCF luciferase reporter activity was also substantially impaired in PRC1-siRNA-treated cells, as previous reported (Figure 3A). PRC1 is a MAP regulator of cytokinesis and could precipitate with KIF23. We further studied the effect of KIF23 on the expression of 11 reported Wnt targets by real-time PCR with specific primers (Supplementary Table 1). KIF23 knockdown significantly inhibited the expression of 9 out of the 11 Wnt targets, and β-catenin or PRC1 knockdown also inhibited most of the 11 Wnt targets (Figure 3B). Western blot analysis confirmed these results and demonstrated that silencing KIF23 markedly suppressed the Wnt/β-catenin signaling pathway by decreasing ABC, β-catenin, p-GSK3β and Wnt target expression in MGC-803 cells, which was partly rescued by LiCl (Figure 3C and Supplementary Figure 5A). PRC1 silencing had the same effects on the Wnt/β-catenin signaling pathway as KIF23 knockdown. However, KIF23 knockdown increased PRC1 expression. Therefore, we combined KIF23 siRNA with β-catenin or PRC1 siRNA to recover the expression of PRC1. Figure 3F showed that ABC, p-GSK3β and Wnt target expression was significantly suppressed after combination of KIF23 siRNA with β-catenin or PRC1 siRNA. Based on the observed effects of KIF23 silencing on activated β-catenin levels, we further investigated the effect on the nuclear distribution of β-catenin. The results obtained from cytoplasmic and nuclear protein extraction and immunofluorescence indicated that KIF23 knockdown impaired the nuclear accumulation of β-catenin (Figure 3D and 3E). Reduction of β-catenin by siKIF23 may also be at the protein level, as it could be blocked by proteasome inhibitor MG132. And KIF23 silencing mediated β-catenin degradation is a proteasome-dependent and ubiquitination-dependent (Supplementary Figure 5B and 5C). Moreover, the inhibition of cell proliferation and colony formation caused by KIF23 siRNA was rescued by LiCl (Figure 3G and 3H), indicating that KIF23 regulates the oncogenic effects of the Wnt/β-catenin signaling pathway. On the other hand, to identify the effect of the Wnt/β-catenin signaling pathway on KIF23, we analyzed the expression of KIF23 after treatment with β-catenin siRNA or LiCl. We found that silencing of β-catenin and activation of the Wnt/β-catenin signaling pathway did not affect the expression and distribution of KIF23 (Supplementary Figure 6).

Considering the effect of KIF23 and PRC1 on the Wnt/β-catenin signaling pathway and cytokinesis, we analyzed the correlation between KIF23 and PRC1 with r2 (r2.amc.nl). The dataset suggested that KIF23 was closely associated with PRC1 and our results showed that PRC1 specifically precipitated with KIF23 (Supplementary Figure 7A and 7B). Furthermore, KIF23 and PRC1 were both found to localize to nuclei (Supplementary Figure 7C). Similar to PRC1, KIF23 was present on the spindle midzone throughout mitosis but was highly enriched at the midbody during cytokinesis, whereas PRC1 remained distributed around the midbody and was not highly concentrated in this region (Supplementary Figure 7D).

### KIF23 activated the Wnt/β-catenin signaling pathway by competitively binding with Amer1

**Figure 4:**
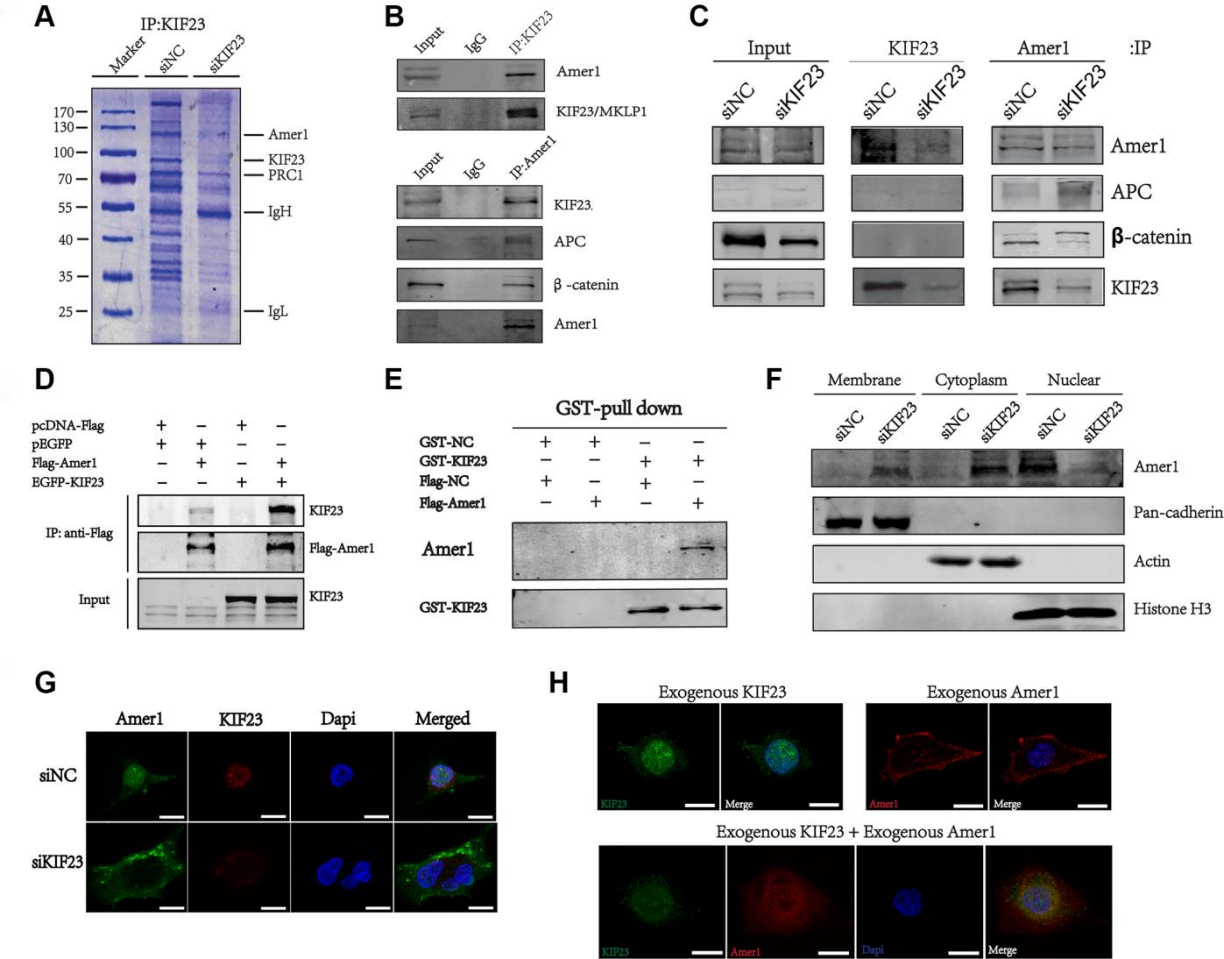
**KIF23 directly interacted with Amer1.** (**A**) Examination of KIF23-binding proteins in MGC-803 cells by IP and SDS PAGE gel Coomassie blue staining before using MS assays. (**B**, **C**) Coimmunoprecipitation analysis between endogenous Amer1 and KIF23. (**D**) Coimmunoprecipitation of FLAG-tagged Amer1 with eGFP-tagged KIF23 in 293T cells as indicated. (**E**) GST-pull down of exogenous Amer1 with KIF23 in MGC-803 cells. GST-NC is the control plasmid of GST-KIF23 and Flag-NC is the control plasmid of Flag-Amer1. Immunofluorescence staining and western blot analysis for the distribution of Amer1 (**F**) and KIF23 (**G**). (**H**) Immunofluorescence images showing the localization of Amer1 in MGC-803 cells after transfection with eGFP-tagged KIF23, FLAG-tagged Amer1, or both.

To our knowledge, no direct target of KIF23 has been reported previously. To identify KIF23 target proteins that may account for the activation of the Wnt/β-catenin signaling pathway, we first used mass spectrometry to identify proteins that bind directly with KIF23, and Amer1 was identified as a putative protein involved in KIF23-mediated activation of the Wnt/β-catenin signaling pathway (Figure 4A and Supplementary Figure 8). To further confirm the interaction between KIF23 and Amer1, we performed coimmunoprecipitation (Co-IP) and GST-pull-down experiments and found that endogenous KIF23 as well as exogenous wild-type KIF23 could be specifically coimmunoprecipitated with Amer1 (Figure 4B–4E). Immunofluorescence staining and Western blotting showed that Amer1 localized to the nuclei instead of the plasma membrane, but transferred to the plasma membrane after KIF23 was silenced in MGC-803 (Figure 4F and 4G). Consistent with the above results, exogenous Amer1 was also enriched in nuclei after cotransfection with a KIF23 overexpression plasmid (Figure 4H).

**Figure 5:**
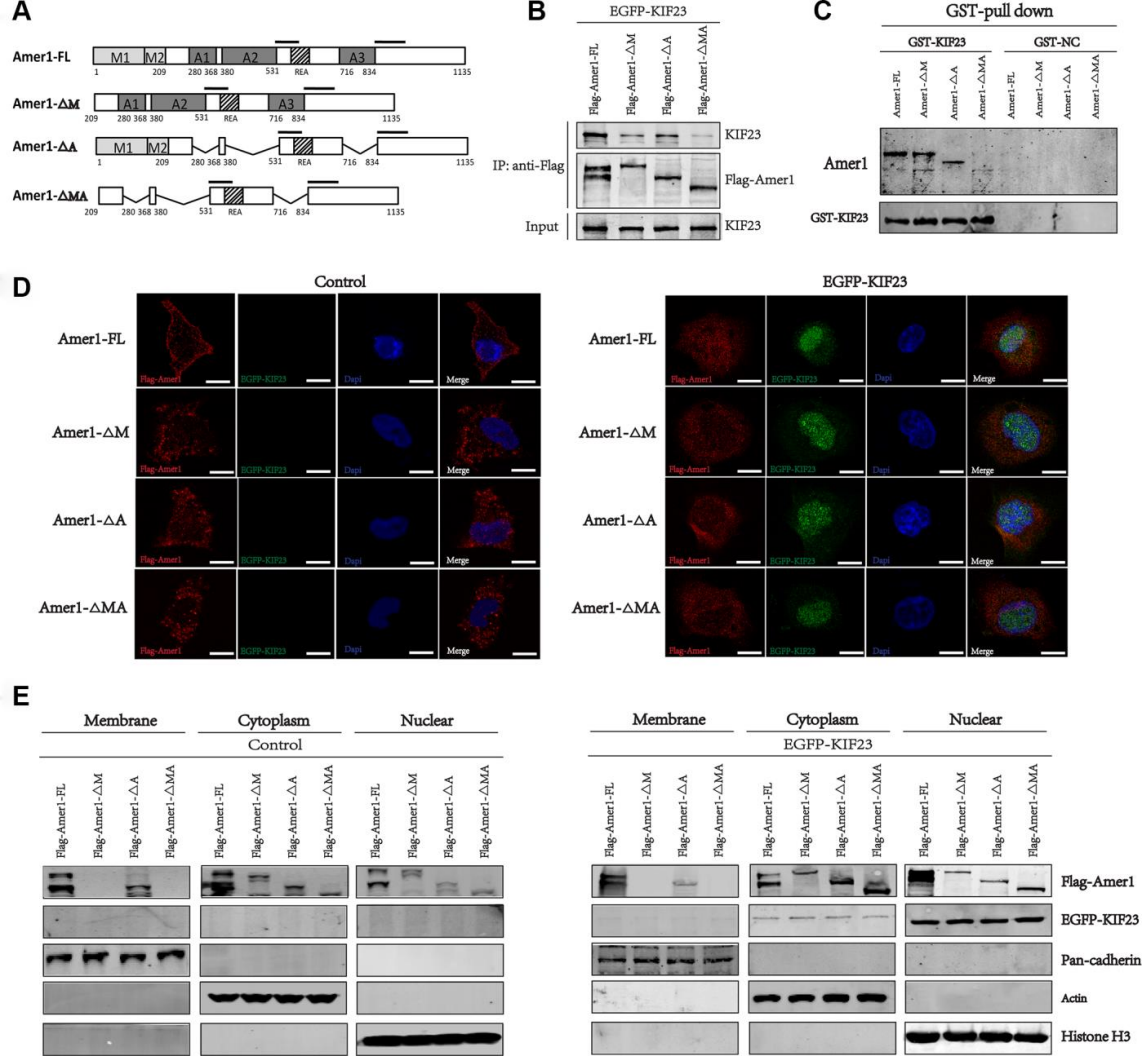
**KIF23 combined with the membrane localization and APC-binding domains within Amer1.** (**A**) Amer1-FL (full length) and its three truncation mutants were constructed, namely, ΔM (lacking the N-terminal membrane localization domains, MLD), ΔA (lacking the APC-binding domains, ABD) and ΔMA (lacking the membrane localization domains and APC-binding domains). Coimmunoprecipitation (**B**) and GST-pull down (**C**) of FLAG-tagged Amer1 with eGFP-tagged KIF23 after transient transfection of 293T cells as indicated. Immunofluorescence staining (**D**) and western blot analysis (**E**) for the distribution of Amer1 and KIF23 in MGC-803 cells after transfection with eGFP-tagged KIF23 or control plasmid and FLAG-tagged Amer1-FL or its three truncation mutants.

Furthermore, we constructed full-length Amer1 and three truncated mutants fused to the FLAG tag and Ds-red fluorescent protein. These constructs were tentatively termed Amer1-FL (full-length Amer1), ∆M (lacking the N-terminal membrane localization domains, MLD), ΔA (lacking the APC-binding domains, ABD) and ΔMA (lacking the membrane localization domains and APC-binding domains) (Figure 5A). Then, Co-IP and GST-pull-down assays were performed, and the results showed that all the Amer1 constructs except Amer1-ΔMA immunoprecipitated with KIF23, suggesting that KIF23 binds with Amer1 within the membrane localization domains and APC-binding domains (Figure 5B and 5C). Notably, cotransfection of the KIF23 plasmid with Amer1-FL led to Amer1 being transferred from the plasma membranes to the nuclei, but the levels of Amer1 in the nuclei were relatively low in the groups cotransfected with the KIF23 plasmid and Amer1-ΔM, ΔA, or, especially ΔMA (Figure 5D and 5E). Thus, the competitive binding of KIF23 with MLD and ABD within Amer1 interfered with the interaction between Amer1 and APC and with the distribution of Amer1, thus attenuating the ability of Amer1 to negatively regulate the Wnt/β-catenin signaling pathway.

### KIF23 promoted proliferation via competitively binding with Amer1 in GC cells and tissues

**Figure 6:**
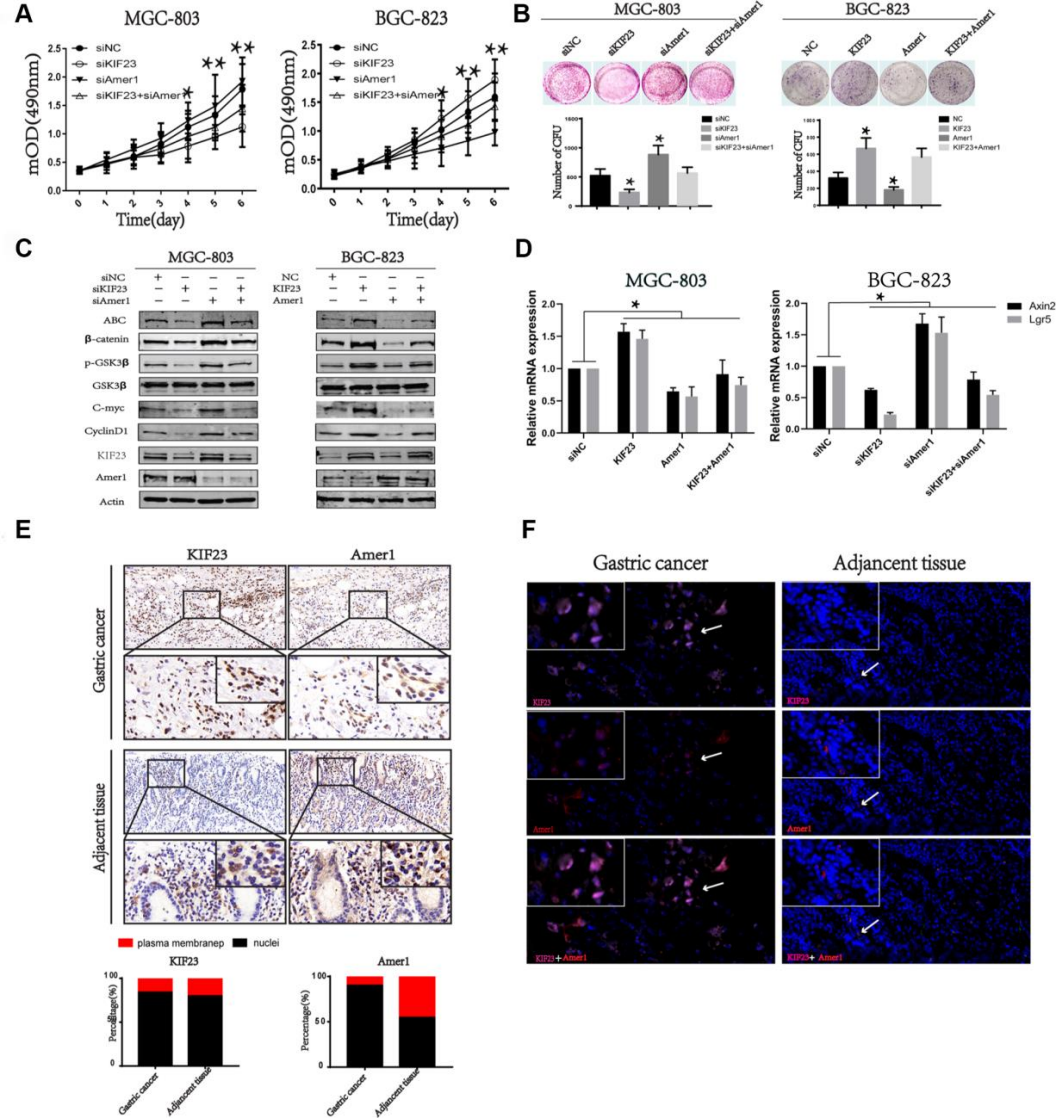
**KIF23 promoted GC cells proliferation via binding with Amer1 and disturbing its distribution.** Effects of cotransfection of KIF23-siRNA and Amer1-siRNA or KIF23and Amer1 overexpression plasmids on cell growth (**A**) and colony formation assay (**B**). (**C**) Western blot analysis of the activity of Wnt/β-catenin signaling pathway and its target proteins after cotransfection of KIF23-siRNA and Amer1-siRNA or KIF23 and Amer1 overexpression plasmids. (**D**) Q-PCR analysis of effects of cotransfection of KIF23-siRNA and Amer1-siRNA or KIF23and Amer1 overexpression plasmids on Wnt targets Axin2 and Lgr5. IHC (**E**) and IF (**F**) were used to analyze the expression and distribution of KIF23 and Amer1 in GC and adjacent normal tissues.

Next, we induced Amer1 knockdown in KIF23 silencing cells, and MTT and colony formation assays indicated that the proliferation of GC cells was reversed. Similarly, KIF23 overexpression promoted cell proliferation, while overexpression of Amer1 reversed the effect (Figure 6A and 6B). Western blot and Q-PCR analysis also indicated Amer1 knockdown reversed the activity of Wnt/β-catenin signaling pathway in KIF23 silencing cells, while overexpression of Amer1 reversed the effects of KIF23 overexpression (Figure 6C and 6D). Finally, the expression and distribution of KIF23 and Amer1 were detected in GC patients by IHC and IF (Figure 6E and 6F). In adjacent tissues, Amer1 mainly localized in the plasma and membrane but it was carried to the nuclei by high expression of KIF23 in GC tissues.

## DISCUSSION

The prognosis of GC remains dismal, and our knowledge of the underlying cellular and molecular pathways that drive GC pathogenesis is limited [14]. In this study, we verified that KIF23 was significantly upregulated in GC tumors and that KIF23 expression was strongly associated with GC progression. We further demonstrated that KIF23 played oncogenic roles in association with the canonical Wnt/β-catenin signaling pathway by targeting Amer1.

KIF23 was abundantly expressed in HCC cells and acted as a novel HCC biomarker [15]. Overexpression of KIF23 in non-small cell lung cancer (NSCLC) governs the crucial functions of KIF23 in regulating the cancerous properties of the cells [16]. KIF23 has been found to increase in GC and silencing KIF23 suppressed cell proliferation recently [5]. Consistently, the public datasets and our experimental results confirmed that KIF23 was upregulated in GC tissues and that expression of KIF23 was relatively high in advanced tumor tissues. Our results indicated KIF23 could regulate the invasiveness of GC cell lines in addition to growth. And silencing of KIF23 increased the percentage of binucleated or multinucleated cells, likely due to a cytokinesis defect, as demonstrated in HeLa cells in previous studies [2, 17].

Although KIF23 has been studied in GC, little was previously known regarding the mechanisms by which KIF23 promotes cancer cell growth. GSEA analysis suggested that KIF23 is closely correlated with the cell cycle, especially the G2/M transition. The Wnt/β-catenin signaling pathway is the major signaling pathway that regulates cancer cell growth and invasion [18, 19]. β-catenin, the central effector molecule in this pathway, is negatively regulated via phosphorylation by a multiprotein complex that includes APC, Axin, GSK-3β, and CK1α [20]. When the Wnt/β-catenin signaling pathway is activated by the binding of Wnt proteins to the LRP/Frizzled complex, the degradation complex is inhibited, limiting the ubiquitin-mediated degradation of β-catenin, which is highly stabilized, leading to the accumulation of this protein in the cytoplasm and subsequent migration to the nucleus [21]. In the nucleus, β-catenin acts as a transcriptional coactivator, binding to the transcription factors of the TCF/lymphoid-enhancing factor (LEF) family to activate the transcription of target genes such as C-Myc and CyclinD1 that regulates cell proliferation and differentiation [22–24]. With regard to its function, we examined the regulation of the Wnt/β-catenin signaling pathway by KIF23 in promoting cell growth. Surprisingly, our data showed that KIF23 activated the Wnt/β-catenin signaling pathway by promoting the accumulation of β-catenin in the nucleus.

PRC1 is a MAP regulator of cytokinesis and has been demonstrated to be an oncogene in HCC [25, 26]. Chen J and Zhan P have demonstrated that PRC1 exerts its oncogenic effect by affecting the destruction complexes to activate the Wnt/β-catenin signaling pathway [27, 28]. It has been reported that PRC1 modulated membrane sequestration of the destruction complex, inhibited APC stability and promoted β-catenin release from the APC complex. And PRC1 could regulate Wnt-regulated-recurrence-associated genes expression including KIF23 [27]. Gruneberg U et al. found PRC1 could precipitate with KIF23 [29]. In our results, silencing PRC1 inhibited the expression of KIF23, while the decreased expression of KIF23 induced the increased expression of PRC1, which might be caused by the compensatory regulation. We confirmed that PRC1 specifically precipitated with KIF23 and these two proteins had similar cellular distribution. And like PRC1, KIF23 also promoted cell proliferation through the Wnt/β-catenin signaling pathway. These results indicated that PRC1 and KIF23 formed a complex to regulate cytokinesis and cell proliferation through Wnt/β-catenin signaling pathway.

To further explore the direct targets of KIF23 in GC cells, proteomic approaches were adopted to identify candidate direct-binding partners of KIF23, and Amer1 was found to be the most likely candidate. Amer1 (APC membrane recruitment 1) is a 1135-amino-acid plasma-membrane-associated protein that is conserved in vertebrates [30, 31]. Structural analysis showed that Amer1 binds to APC and acts as an inhibitor of the Wnt/β-catenin signaling pathway [32]. However, the relationship between KIF23 and Amer1 has not been characterized to date. Considering the negative regulatory role of Amer1 in the Wnt/β-catenin signaling pathway, we hypothesized that KIF23 might competitively bind to the APC-binding and membrane localization domains within Amer1, and then, KIF23 carries Amer1 to the nucleus to suppress the inhibitory effects of this protein on the Wnt/β-catenin signaling pathway at the membrane. This model was supported by the observation that silencing KIF23 increased the association of Amer1 with APC and led to localization of Amer1 to the membrane.

## CONCLUSIONS

**Figure 7:**
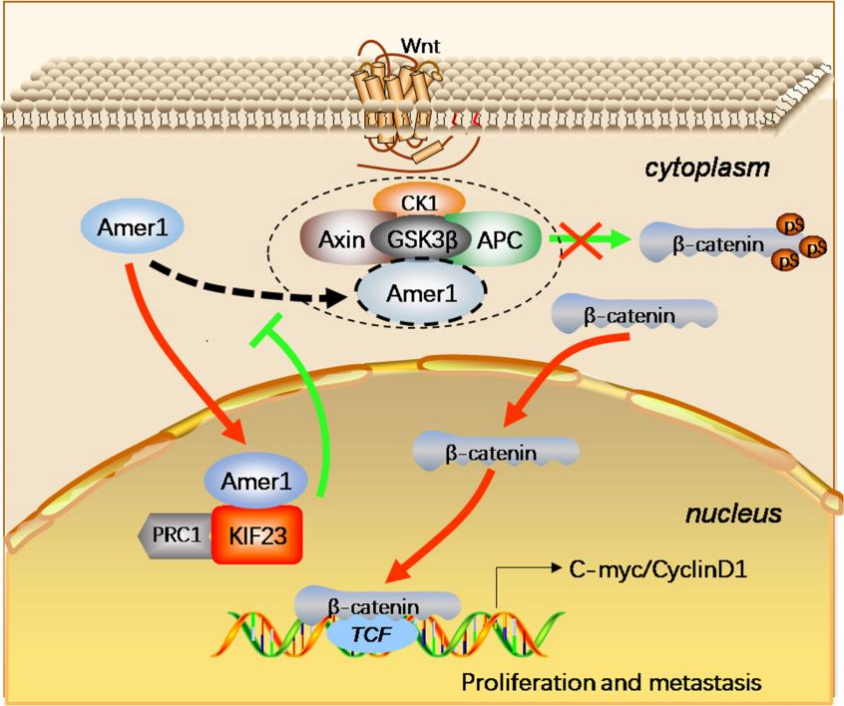
**Schematic figure represented the functions of KIF23 in the Wnt/β-catenin signaling pathway in GC.** The KIF23 overexpression exhibited competitive binding with Amer1 and carried it to the nuclear to block the association of Amer1 with APC, thus attenuating the ability of Amer1 to negatively regulate Wnt/β-catenin signaling, resulting in activation of this signaling pathway.

In conclusion, KIF23 regulates the expression and localization of β-catenin to potentiate the Wnt/β-catenin signaling pathway by competitively binding with Amer1, blocking the binding of Amer1 with APC and perturbing the distribution of Amer1 (Figure 7). Thus, our data indicate that KIF23 is a novel oncogene that drives GC occurrence and development.

## MATERIALS AND METHODS

### Human GC samples

Twelve pairs of gastric tumor tissues were obtained from patients with GC treated at Xinhua Hospital, Shanghai Jiao Tong University School of Medicine, China, between 2009 and 2014. The GC tissues for IHC containing 4 normal samples and 118 matched pairs of adjacent tissue and tumors were obtained from the Shanghai Biochip Company (Supplementary Table 3). All patients were diagnosed by pathological analyses based on the TNM criteria defined by the International Union Against Cancer (UICC). The study protocol conformed to the ethical guidelines of the Declaration of Helsinki and was approved by the Institutional Review Board and Ethics Committee of Xinhua Hospital.

### Cell lines and culture

The HEK293T and human GC cell lines (SGC-7901, MGC-803, BGC-823, and MKN-45) were purchased from the Chinese Academy of Sciences Cell Bank of Type Culture Collection. Cells were maintained in DMEM containing 10% FBS supplemented with 100 U/ml penicillin and 100 μg/ml streptomycin (Gibco, USA). For tumor sphere cultures, cells were seeded in dishes precoated with 18 mg/ml polyHEMA and cultured in serum-free DMEM/F12 media supplemented with 20 ng/ml EGF, 10 ng/ml bFGF, 1% N2 and 2% B27. For cell synchronization, MGC-803 cells treated with siRNAs were cultured twice with 2 mM thymidine for 16-24 h and once with drug-free fresh medium and then released into fresh medium for 12-16 h. After release, the medium was replaced with free medium containing 10 ng/ml nocodazole.

### Cell transfection

The siRNAs, including those targeting KIF23, PRC1, β-catenin and Amer1, were designed and synthesized by the Shanghai Sangon Company. The following plasmid constructs were used: M50 Super 8×TOPFlash and M51 Super 8×FOPFlash were gifts from Randall Moon (Addgene); pEGFP-C1-MKLP1 was a gift from Masanori Mishima; Renilla luciferase-Pol III was a gift from Norbert Perrimon; and the EGFP fusion expression vector pEGFP-C1 was obtained from Clontech. The plasmids harboring the Amer1-FL, ΔM, ΔA, ΔMA, KIF23 shRNAs (sh951, sh703) and the respective control vectors were provided by Shanghai GeneChem Co., Ltd. (Shanghai, China). Lipofectamine 3000 (Life Technologies, Carlsbad, CA, USA) was used for plasmid transfection.

### Cell viability and adhesion-dependent colony formation assay

GC cells were seeded in a 96-well plate at 1500-3000 cells per well for 0-6 days, and cell viability was detected with 3-(4,5-dimethyl-2-thiazolyl)-2,5-diphenyl-2-H-tetrazolium bromide (MTT) (Sigma-Aldrich). The optical density at 490 nm was measured in a multiwell plate reader (FLX800, Bio-TEK). GC cells were plated in 60-mm dishes at a density of 2×10^3^ cells per well for the adhesion-dependent colony formation assay. The culture medium was changed every 3-4 days. Then, 3-4 weeks later, the remaining colonies were fixed with 4% paraformaldehyde and stained with crystal violet. The colonies were counted according to the defined colony size.

### Flow cytometry and cell cycle analysis

Cell cycle analysis was performed using the BD Cycletest Plus DNA Reagent Kit and FITC Brdu Flow Kit (BD Pharmagen, USA) following the manufacturer’s protocol. Multi-color FACS analysis was performed using FACS Canton II, and analyzed by FlowJo software.

### Apoptosis assay

Apoptosis was measured using the FITC Annexin V Apoptosis Detection Kit I (BD Pharmagen, USA) following the manufacturer’s protocol, as previously described [33]. Cells were analyzed by a FACS Canto II flow cytometer (BD Biosciences, USA).

### RNA extraction and quantitative real-time PCR

Total RNA was extracted using TRIzol reagent (Invitrogen, USA) according to the manufacturer’s instructions. The concentration and quality of the total RNA were assessed with a NanoDrop spectrophotometer (Thermo Fisher Scientific, USA). For mRNA expression analysis, reverse transcription was performed using PrimeScript RT master mix (TaKaRa, Japan). Quantitative real-time PCR analysis was performed in triplicate on a 7900 HT real-time PCR system (Applied Biosystems, USA) using SYBR Premix Ex Taq (TaKaRa, Japan), and the expression level of ACTIN was used as an endogenous control. The results were analyzed using the 2^–ΔΔct^ calculation method. The primers used for the experiments are listed in Supplementary Table 1.

### Nano-LC MS/MS analysis

Fifty microgram protein samples were submitted for proteomic analysis using Nano-LC MS/MS. Experiments were performed on a QExactive mass spectrometer coupled to an Easy nLC (Thermo Fisher Scientific) at Shanghai Applied Protein Technology Co., Ltd.

### In vitro cell migration and invasion assay

Cell migration and invasion assays were conducted in 24-well Transwell cell chambers with 8-μm pores as previously described [34].

### Nuclear protein extraction and cell fractionation

Cell nuclear and cytoplasmic proteins were extracted using nuclear and cytoplasmic extraction reagents (Thermo, USA). Cells were treated with the CER I:CER II:NER reagents at 200:11:100 μl, and nuclear and cytoplasmic proteins were then extracted separately. Cell membrane proteins were extracted using the Mem-PER Plus Membrane Protein Extraction Kit (Thermo, USA) according to the manufacturer’s protocol.

### Western blot analysis

The cells were lysed in equal volumes of ice-cold lysis buffer with a protease inhibitor cocktail. Cell lysates were separated by SDS-PAGE and then transferred to a 0.2-μm PVDF membrane (Bio-Rad, USA). After blocking with Odyssey blocking buffer (Li-COR Biosciences, USA), the membrane was incubated with primary antibody (1:1000) at 4°C overnight, followed by incubation with IRDye 800CW or 680 secondary antibody (1:5000, LI-COR Biosciences, USA). Actin was used as an endogenous control. An Odyssey infrared imaging system was used to visualize the targeted protein bands. All antibodies used in the experiments are listed in Supplementary Table 2

### Coimmunoprecipitation

MGC-803 or HEK293T cells were transfected with or without siRNA or plasmid for 48 hours, and 100 μg of protein extract was diluted to1 ml in Co-IP buffer (Thermo, USA); then, 2 μg of KIF23 (Santa Cruz Biotechnology, USA) or FLAG (Cell Signaling Technology, USA) antibody was added to the protein samples, and the mixtures were incubated overnight at 4°C with rotation. Twenty microliters of 50% protein A/G-agarose bead slurry (equilibrated in Co-IP buffer) was added, and after 2 h of incubation at 4°C, the beads were washed 4 times with Co-IP buffer (1 ml per wash) and diluted with 1×loading buffer. Western blotting was performed, and β-catenin (Santa Cruz Biotechnology, USA), Amer1 (Abcam, USA), and APC (Cell Signaling Technology, USA) were detected.

### GST-pull down assay

GST-pull down assay was performed with a Pierce^TM^ GST Protein Interaction Pull-Down Kit (Thermo, USA) following the manufacturer’s protocol. In brief, we first prepared GST-tagged bait protein from the *E. coli* expression system. Then, prey protein was added to the Pierce Spin Column immobilized with bait protein. Finally, Western blotting was performed and KIF23 and Amer1 were detected.

### Immunofluorescence assay

Specimens were prepared as previously described. Images were captured using a Leica SP5 laser scanning confocal microscope [35].

### Luciferase reporter assay

Transcriptional activity assays were performed using the Luciferase Reporter Assay System (Promega, USA) according to the manufacturer’s instructions. Briefly, plasmids or siRNAs were transfected into cells using Lipofectamine 3000. After culture for certain duration, the cells were collected, and relative luciferase activity was detected using a luminometer (Promega GloMax 20/20, USA).

### In vivo xenograft and treatment experiments

For in vivo studies, 4-6-week-old male nude mice were purchased from the Shanghai Laboratory Animal Center of China. Treated or untreated MGC-803 cells (2×10^6^ cells in 200μl of 1×PBS) were subcutaneously injected in to the right flanks of nude mice to establish tumors. All animal procedures were carried out with the approval of the Institutional Committee of Shanghai Jiao Tong University School of Medicine for Animal Research.

### Immunohistochemical staining

Specimens were prepared as previously described [35]. Automated image acquisition was performed using an AperioScanScope XT slide scanner system with a 20× objective (Aperio Technologies).

### Bioinformatics analysis

The expression patterns of KIF23 in GC specimens were analyzed based on the public datasets GEO (GSE2685, GSE65801 and GDS4198) and TCGA. GSEA was carried out using GSEA software. The top 32 and bottom 32 gastric tissues from the public dataset (GSE65801) were scored according to KIF23 expression for GSEA analysis.

### Statistical analysis

Statistical significance between groups was determined by two-tailed Student’s t-test and one-way ANOVA. Differences were considered to be significant when P<0.05. All statistical data are shown as the means ± standard deviations (SDs) and were analyzed for statistical significance with GraphPad Prism 5.0 for Windows (GraphPad Software, USA).

### Ethics approval

The study protocol conformed to the ethical guidelines of the Declaration of Helsinki and was approved by the Institutional Review Board and Ethics Committee of Xinhua Hospital. Informed consent was obtained from each individual enrolled in this study.

## Supplementary Material

Supplementary Figures

Supplementary Tables
