# Supplementary material for: KIF23 activated Wnt/β-catenin signaling pathway through direct interaction with Amer1 in gastric cancer
Source: Aging (Albany NY). 2020 May 4;12(9):8372–96. doi: 10.18632/aging.103146 (PMC7244035; doi:10.18632/aging.103146)
Supplement: Supplementary Figures [file aging-12-103146-s002..pdf]

SUPPLEMENTARY FIGURES

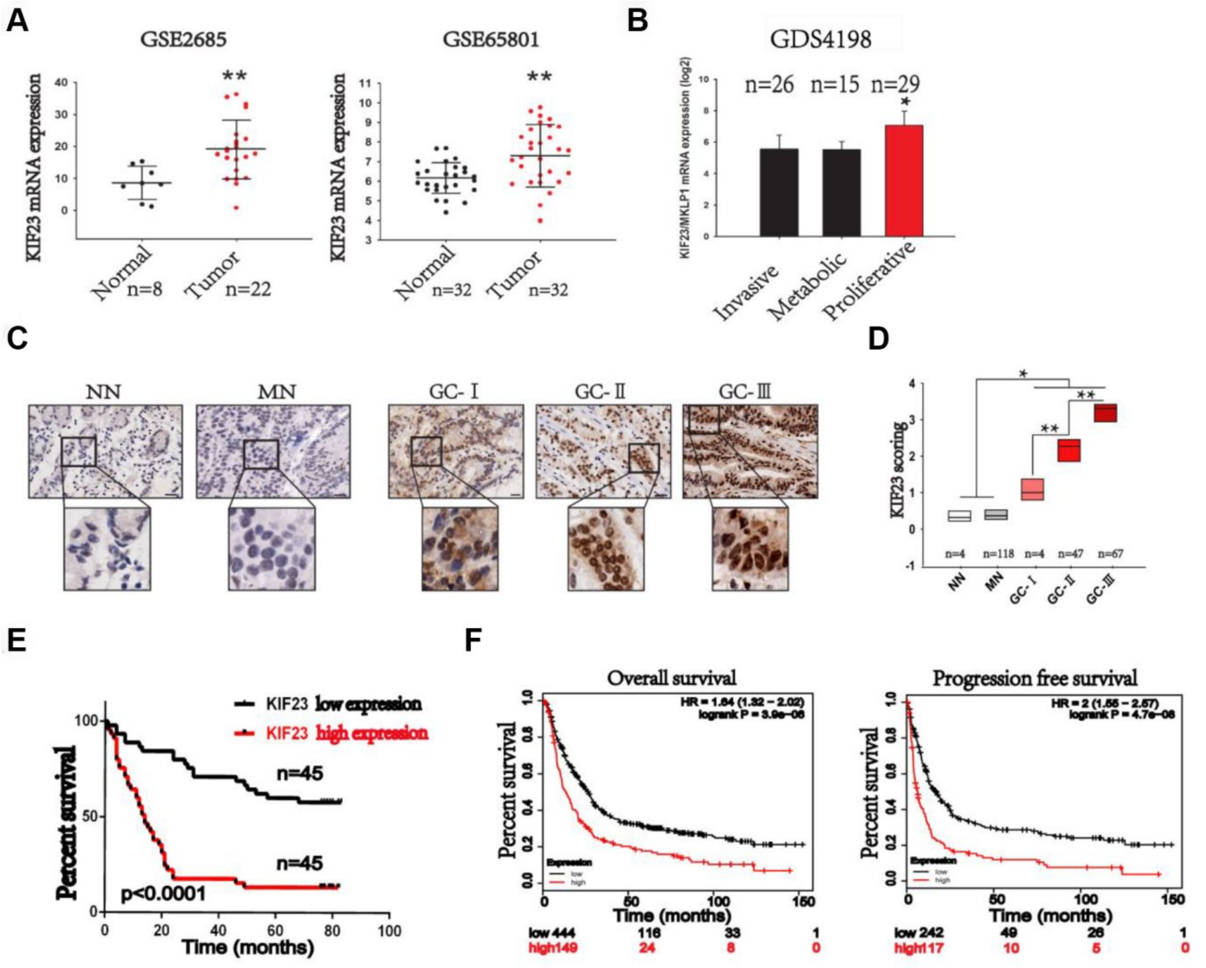

Supplementary Figure 1. KIF23 had high expression in GC and associated with the survival time. (**A**) GEO dataset analysis of KIF23 mRNA level in GC. (**B**) Analysis of KIF23 gene expression in different types of GC from GEO dataset (GDS4198). (**C**) Representative immunohistochemistry (IHC) staining for KIF23 expression in normal (NN), matched normal (MN) and GC tissues, and (**D**) KIF23 expression intensity scores for 118 pairs of tissues. (**E**) Survival analysis was performed to establish the relationship between KIF23 expression levels and the survival of GC patients. (**F**) Using publicly available datasets to analyze overall survival and progression-free survival of patients exhibiting high or low KIF23 expression. Data are the means ± SEMs of three independent experiments. * $p < 0.05$, ** $p < 0.01$, *** $p < 0.001$ vs. control.

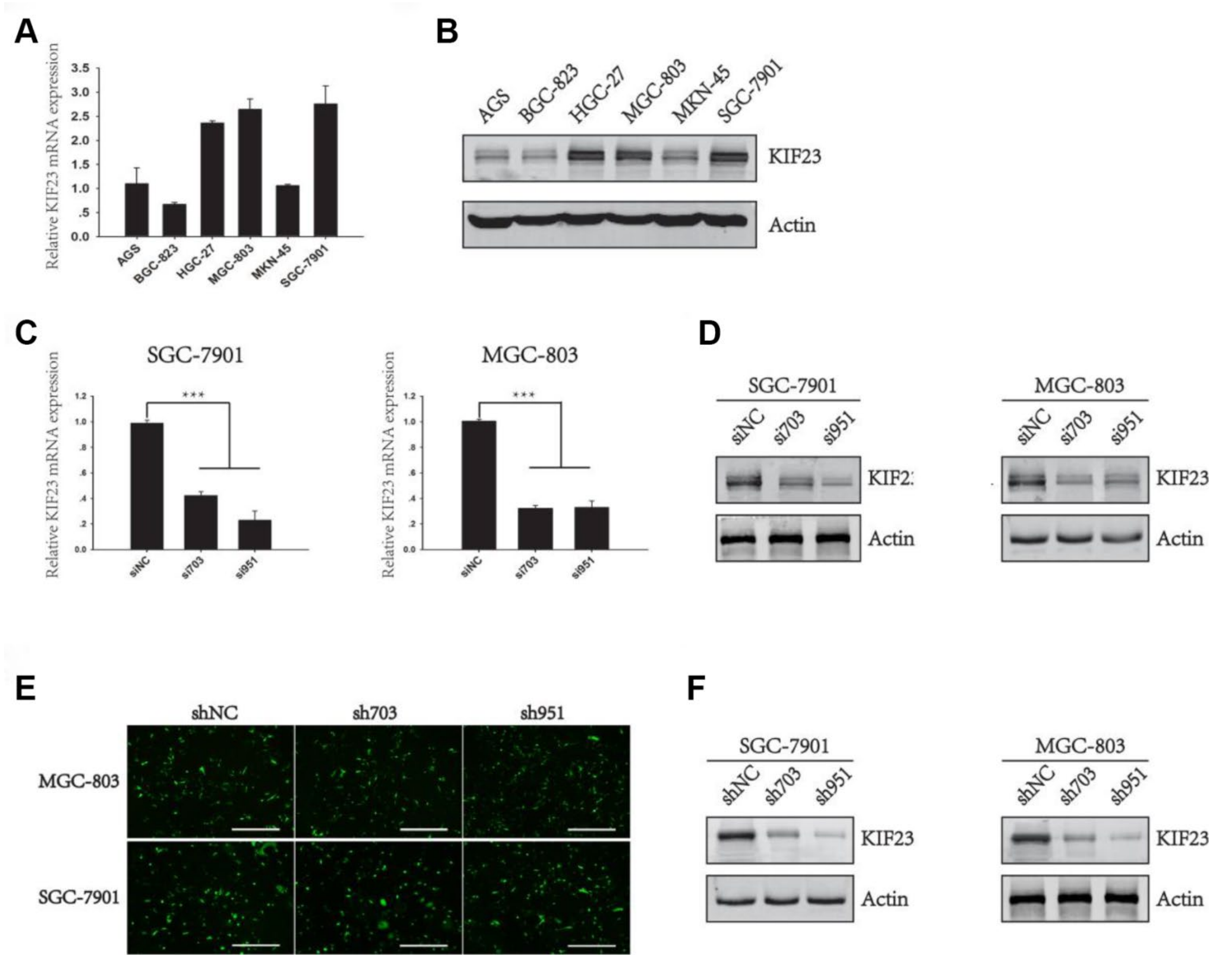

Supplementary Figure 2. Expression of KIF23 in 6 GC cell lines and analysis of the expression of KIF23 in cells with siRNA and shRNA. The mRNA (**A**) and protein levels (**B**) of KIF23 in 6 GC cell lines. Q-PCR (**C**) and Western blot (**D**) analysis of the silencing effects of siRNA-KIF23 in SGC-7901 and MGC-803 cells. (**E**) Transfection efficiency of shRNA-KIF23 in SGC-7901 and MGC-803 cells. (**F**) Protein levels of KIF23 in SGC-7901 and MGC-803 cells transfected with shRNAs.

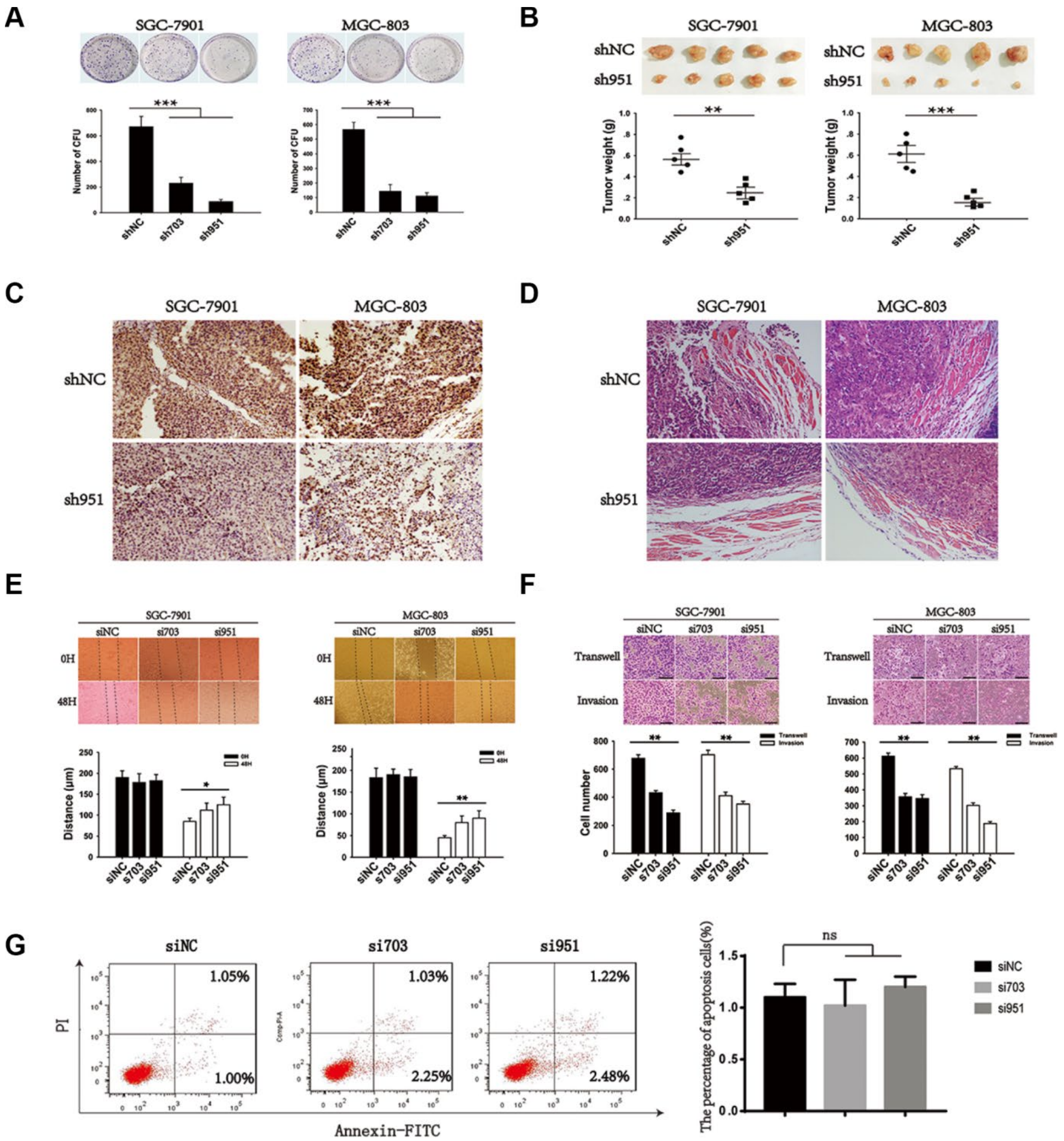

Supplementary Figure 3. Effects of silencing KIF23 on the growth and invasion behavior of GC cells. (**A**) Deletion of KIF23 to observe its effect on colony formation capacity in SGC-7901 and MGC-803 cells. Representative photographs of the colonies and the statistical analysis are shown. (**B**–**D**) In vivo tumorigenicity of GC cells with KIF23 knocked down. (**B**) Tumors were separated (top), weighed and statistically analyzed (bottom). (**C**) IHC staining for KIF23 was completed in tumors formed in both the control group and KIF23 knockdown group. (**D**) HE staining was used to analyze whether tumors of the control group and KIF23 knockdown group invaded the muscle tissues. (**E**) Analysis of wound healing at 0 and 48 h after scratching using ImageJ software. (**F**) Deletion of KIF23 to determine its role in GC cell migration and invasion. Representative photograph of migrated cells and the statistical analysis are shown. (**G**) Flow cytometric analysis of cell apoptosis in GC cells transfected with siRNAs.* $p < 0.05$, ** $p < 0.01$, *** $p < 0.001$. ns, no significant difference.

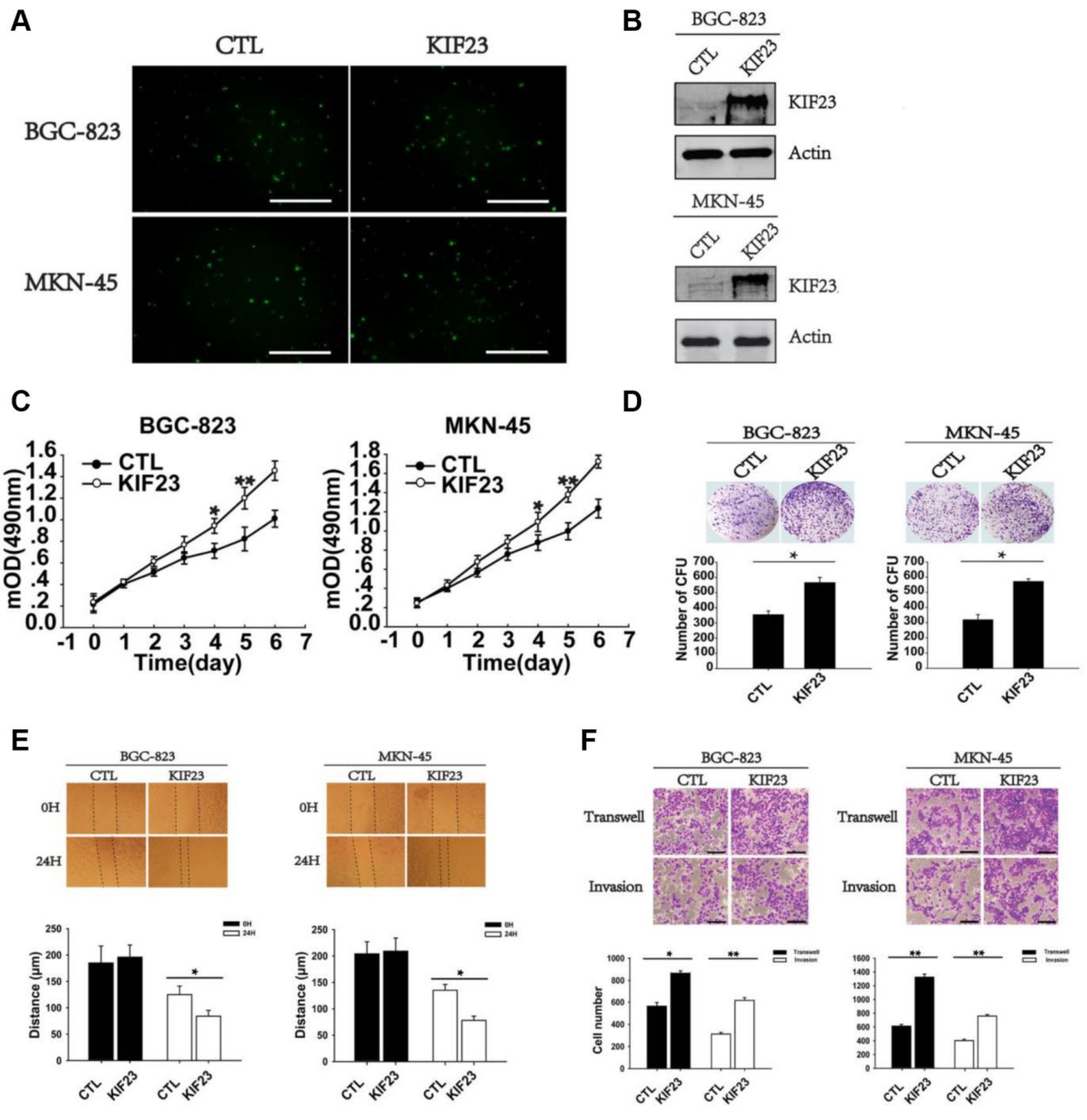

Supplementary Figure 4. Effects of KIF23 overexpression on the growth and invasion behavior of GC cells. (**A**) Transfection efficiency and (**B**) protein expression of KIF23 in MKN-45 and BGC-823 cells transfected with the KIF23 overexpression plasmid. (**C**) MTT assay and (**D**) colony formation experiments were performed. (**E**) Analysis of wound healing at 0 and 48 h after scratch wounding, using ImageJ software. (**F**) GC cell migration and invasion assays were performed after overexpression of KIF23. Representative photograph of migrated cells and the statistical analysis are shown.

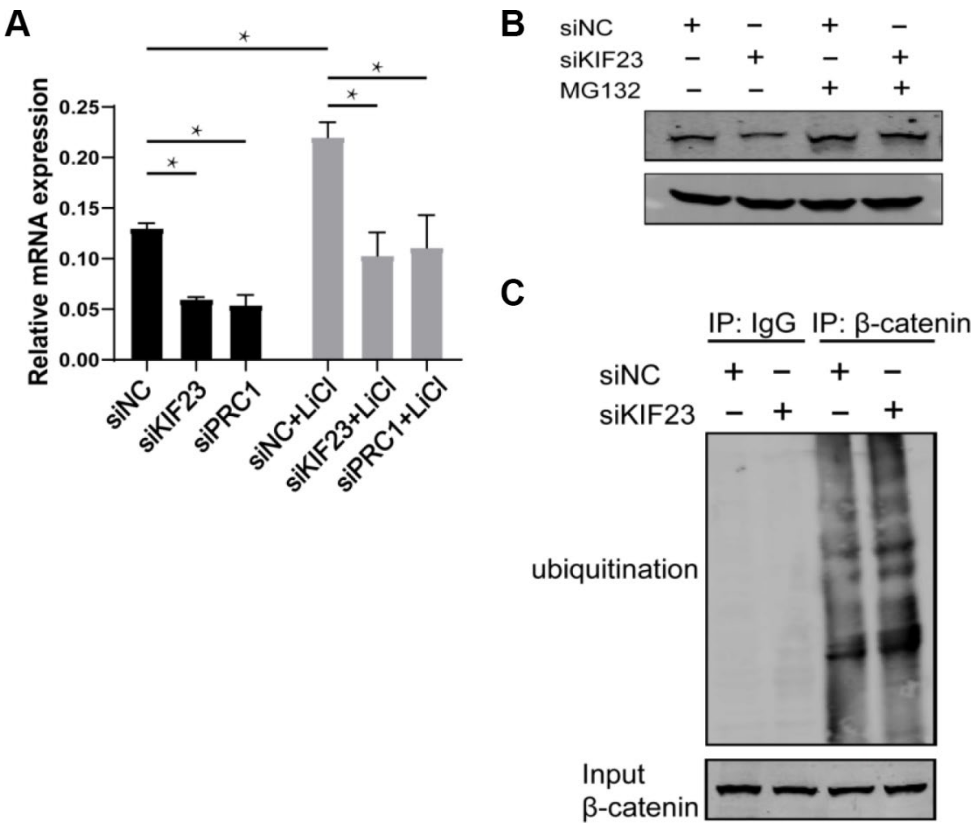

Supplementary Figure 5. Effects of silencing KIF23 on β-catenin level. (**A**) Q-PCR analysis of siKIF23 or siPRC1 and LiCl treatment on the expression of β-catenin. (**B**) Western blot analysis of β-catenin in MGC-803 cells treated with siNC or siKIF23 before and after additional MG132 (20uM) treatment for 6h. (**C**) Silencing KIF23 increased the level of ubiquitination of β-catenin. MGC-803 cells were treated with siNC or siKIF23 for 48h before immunoprecipitation and western blot.

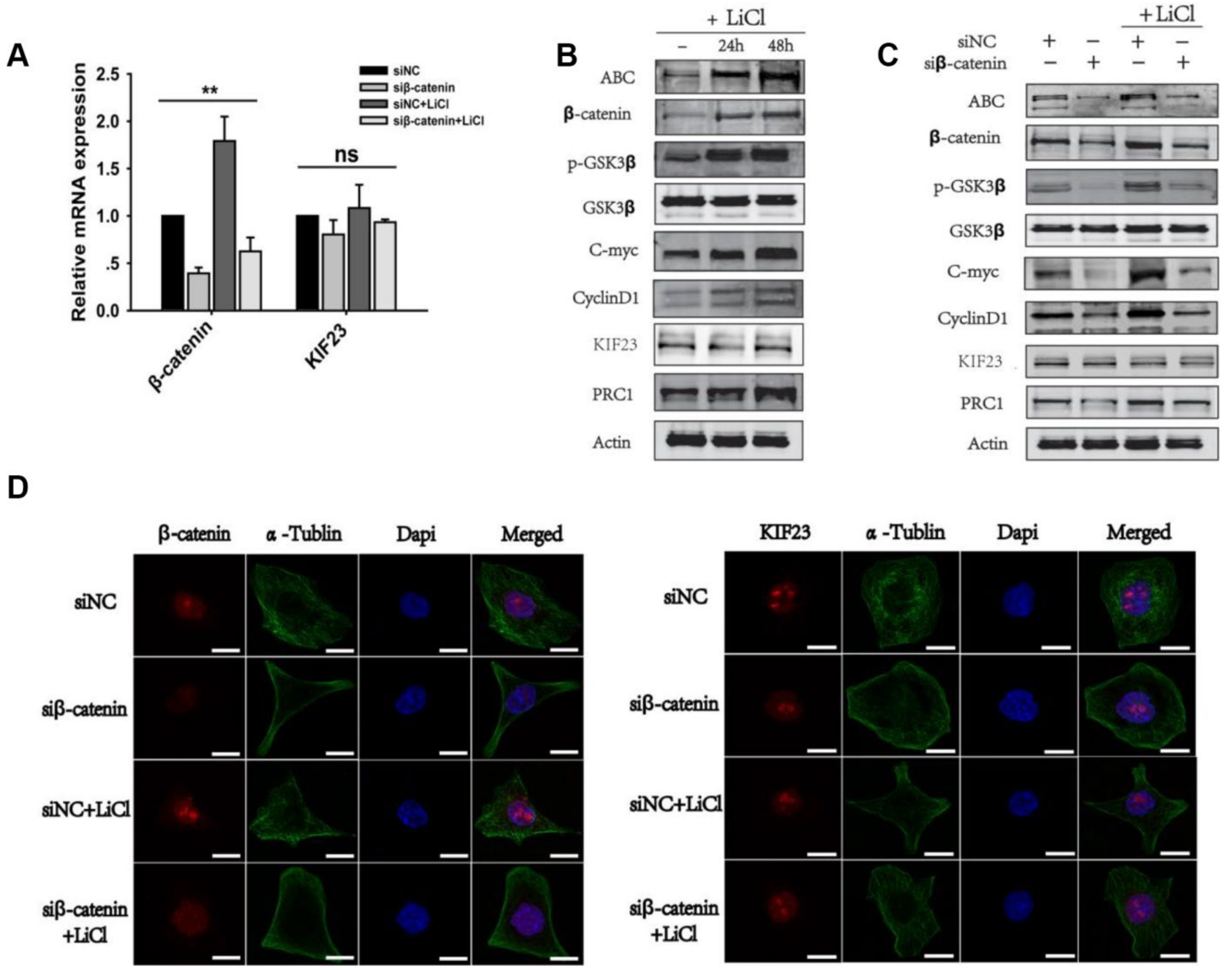

Supplementary Figure 6. KIF23 was not regulated by the Wnt/β-catenin signaling pathway. (**A**) Q-PCR analysis of the effect of the Wnt/β-catenin signaling pathway on KIF23. (**B**) Western blot analysis of the key proteins in the Wnt/β-catenin signaling pathway after treatment with LiCl. (**C**) Effect of inhibition or activation of Wnt/β-catenin on the expression of KIF23. (**D**) Immunofluorescence images showing the expression of β-catenin and KIF23 in MGC-803 cells transfected with siRNAs.

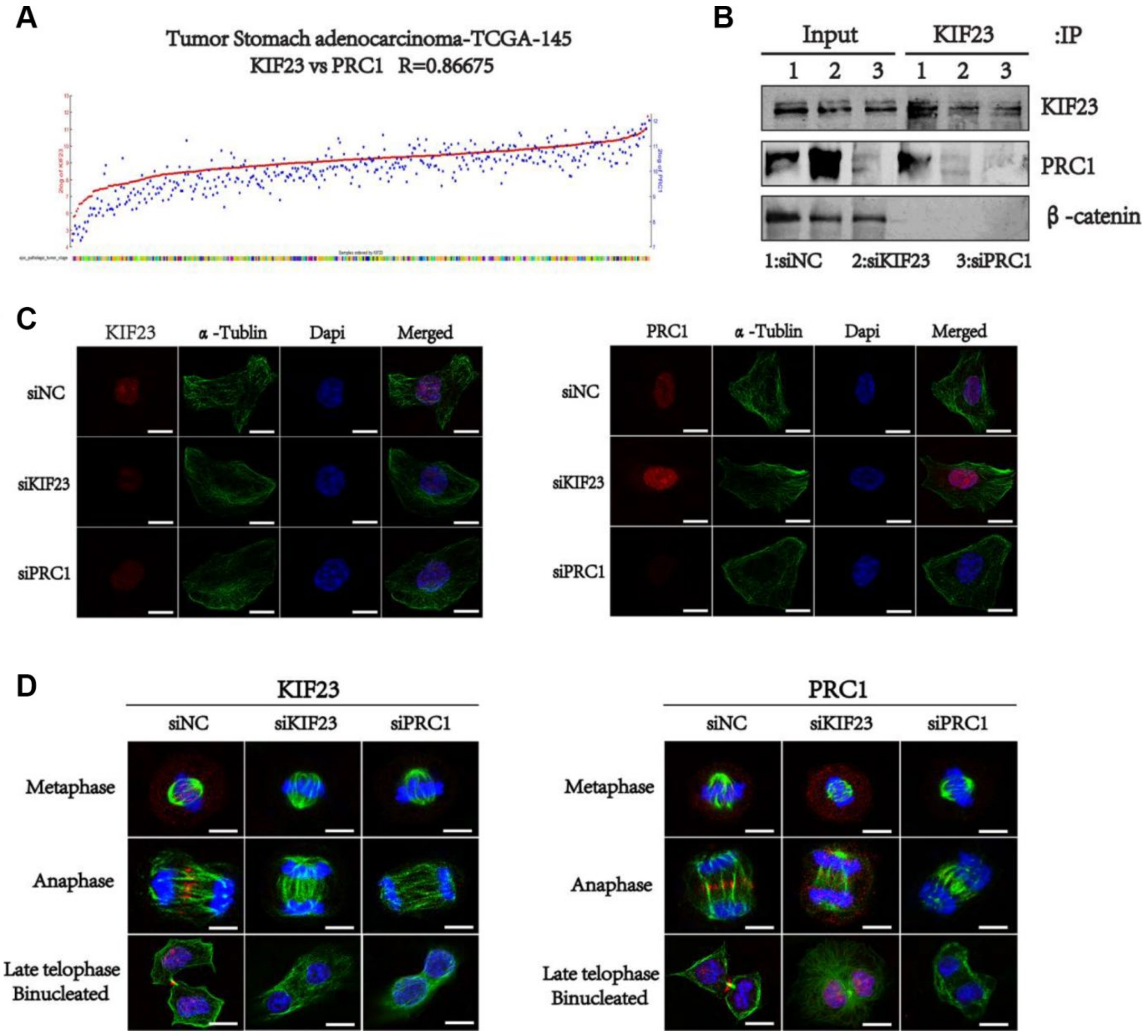

Supplementary Figure 7. The relationship between KIF23 and PRC1. (**A**) TCGA dataset analysis of the correlation between KIF23 and PRC1 with r2 (r2.amc.nl). (**B**) Coimmunoprecipitation analysis of the interaction between PRC1 and KIF23. (**C**) Immunofluorescence staining for KIF23 and PRC1 after siRNAs treatment. (**D**) MGC-803 cells treated with siRNAs for 48 h were synchronized at different times by using thymidine and nocodazole and stained for KIF23 and PRC1. DNA was stained with DAPI. Bar, 10μm.

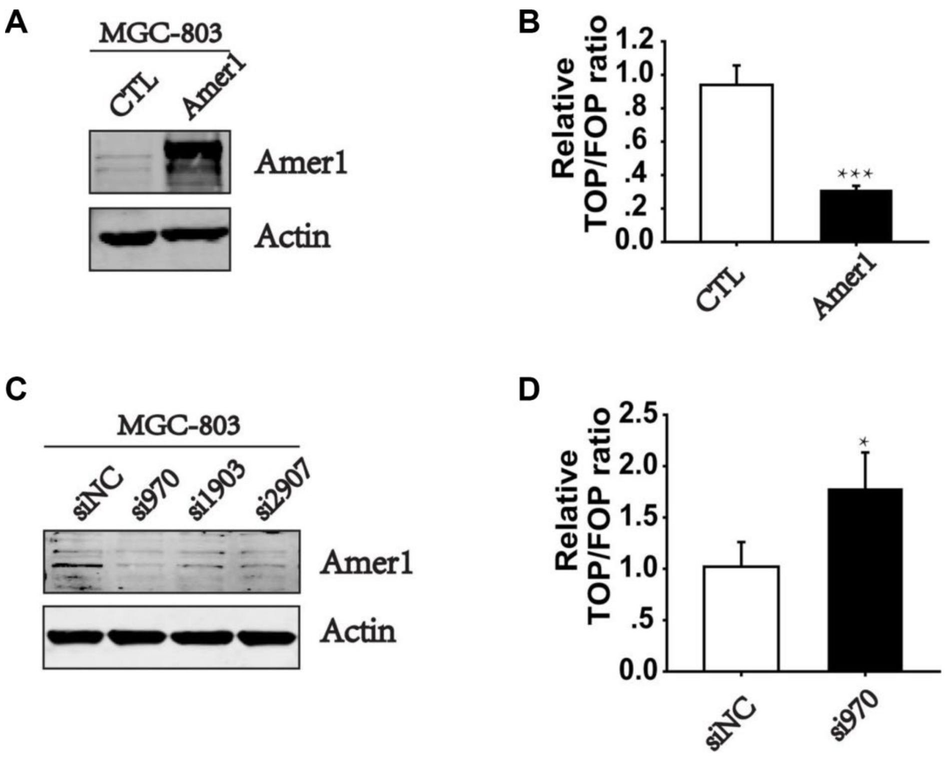

Supplementary Figure 8. Amer1 negatively regulated the Wnt/β-catenin signaling pathway. (**A**) Analysis of protein expression of Amer1 in MGC-803 cells transfected with the Amer1 plasmid. (**B**) Luciferase reporter assay analysis of the proportions of TOP and FOP in MGC-803 cells transfected with the Amer1 plasmid. (**C**) Western blot analysis of the expression of Amer1 in MGC-803 cells transfected with siRNAs. (**D**) Changes in endogenous Wnt/TCF reporter activity in MGC-803 cells after Amer1 silencing. All experiments were repeated at least three times. * $p < 0.05$, ** $p < 0.01$, *** $p < 0.001$.
