# Supplementary material for: KIF23 activated Wnt/β-catenin signaling pathway through direct interaction with Amer1 in gastric cancer
Source: Aging (Albany NY). 2020 May 4;12(9):8372–96. doi: 10.18632/aging.103146 (PMC7244035; doi:10.18632/aging.103146)
Supplement: Supplementary Tables [file aging-12-103146-s001..pdf]

SUPPLEMENTARY TABLES

Supplementary Table 1. Primers used in study.

Name	Forward sequence (5'-3')	Reverse sequence (5'-3')
ACTIN	TGAAGTACCCCATCGAGCACGGCA	GATAGCACAGCCTGGATAGCAACG
SURVIVIN	CTTGGCCCAGTGTTTCTTCT	TCTCCGCAGTTTCCTCAAAT
PRC1	GAGAGCTCTGGGACAGGTTG	TCTTCCAACCGATCCACTTC
C-MYC	CACATCAGCACAACTACG	TTCGCCTCTTGACATTCT
cyclinD1	CTCGGTGTCCTACTTCA	TCCTCGCACTTCTGTTC
β-catenin	GTGTGGCGACATATGCAGCT	CAAGATCAGCAGTCTCATTC
DUT	TTGATGTAGGAGCTGGTGTCA	GCAAATGAGCTGTGCAATTC
MMP7	GTCTCGGAGGAGATGCTCAC	GGAATGTCCCATACCCAAAG
LGR5	TGTGCATTTGGAGTGTGTGA	CACGTTCATCTTGAGCCTGA
TCF1	CCCACAGGTGATGAGCTACC	GCTCCTCCTTGCTAGGGTTC
LEF1	ATATGATTCCCGGTCCTCCT	TGAGGCTTCACGTGCATTAG
DKK1	CATCAGACTGTGCCTCAGGA	GTCCATGAGAGCCTTTTCTCC
POLD3	CTTGGTGTCTGGCAGTCTCA	TACACATGGATGCTGGCAGT
AXIN2	AGTCAGCAGAGGGACAGGAA	GTGGACACCTGCCAGTTTCT
KIF23	TCCCTGTTGACTTTGGGAAG	TGTACGCCCTCCAAGAGAAT

Supplementary Table 2. Antibodies used in study.

Antibody	Source	Country
ABC	Cell Signaling Technology	USA
β-catenin	Cell Signaling Technology	USA
p-GSK3β	Cell Signaling Technology	USA
GSK3β	Cell Signaling Technology	USA
C-myc	Cell Signaling Technology	USA
CyclinD1	Cell Signaling Technology	USA
IRDye 800CW	LI-COR Biosciences	USA
IRDye 680CW	LI-COR Biosciences	USA
KIF23	Santa Cruz Biotechnology	USA
FLAG	Cell Signaling Technology	USA
Amer1	Abcam	USA
APC	Abcam	USA
PRC1	Abcam	USA
Histon H3	Beyotime	China
Pan-cadherin	Abcam	USA
Actin	Cell Signaling Technology	USA

Supplementary Table 3. Summary of patient characteristics.

Clinicopathological feature	Number of cases	Expression of KIF23 (mean±SEM)	P value
Gender			
Male	79	1.0792±0.464	0.177
Female	39	0.9432±0.468	
Age(years)			
≥60	75	0.8727±0.454	0.274
<60	43	1.0467±0.485	
Tumor size(cm)			
≥5	69	1.8322±0.546	0.0412*
<5	49	0.5667±0.213	
Tumor venous infiltration			
Yes	43	1.5612±0.512	0.0481*
No	0		
Not available	75	0.6182±0.232	
TNM stage			
I	4	0.9723±0.572	
II	47	2.2123±0.612	0.00792**[a]
III	67	3.3465±0.523	0.00537**[b]

*P<0.05,**P<0.01 versus the control.a,b, versus the stage I.
